# Hypoxia-Induced miR-15a Promotes Mesenchymal Ablation and Adaptation to Hypoxia during Lung Development in Chicken

**DOI:** 10.1371/journal.pone.0098868

**Published:** 2014-06-02

**Authors:** Rui Hao, Xiaoxiang Hu, Changxin Wu, Ning Li

**Affiliations:** 1 State Key Laboratory for Agrobiotechnology, China Agricultural University, Beijing, P. R. China; 2 College of Animal Science and Technology, China Agricultural University, Beijing, P. R. China; 3 College of Animal Science, Yunnan Agricultural University, Kunming, P. R. China; University of Pécs Medical School, Hungary

## Abstract

The lungs undergo changes that are adaptive for high elevation in certain animal species. In chickens, animals bred at high elevations (e.g., Tibet chickens) are better able to hatch and survive under high-altitude conditions. In addition, lowland chicken breeds undergo physiological effects and suffer greater mortality when they are exposed to hypoxic conditions during embryonic development. Although these physiological effects have been noted, the mechanisms that are responsible for hypoxia-induced changes in lung development and function are not known. Here we have examined the role of a particular microRNA (miRNA) in the regulation of lung development under hypoxic conditions. When chicks were incubated in low oxygen (hypoxia), miR-15a was significantly increased in embryonic lung tissue. The expression level of miR-15a in hypoxic Tibet chicken embryos increased and remained relatively high at embryonic day (E)16–20, whereas in normal chickens, expression increased and peaked at E19–20, at which time the cross-current gas exchange system (CCGS) is developing. *Bcl-2* was a translationally repressed target of miR-15a in these chickens. miR-16, a cluster and family member of miR-15a, was detected but did not participate in the posttranscriptional regulation of *bcl-2*. Around E19, the hypoxia-induced decrease in Bcl-2 protein resulted in apoptosis in the mesenchyme around the migrating tubes, which led to an expansion and migration of the tubes that would become the air capillary network and the CCGS. Thus, interfering with miR-15a expression in lung tissue may be a novel therapeutic strategy for hypoxia insults and altitude adaptation.

## Introduction

The cross-current gas exchange system (CCGS) and blood-gas barrier (BGB) morphology are two important factors that determine the avian lung air-diffusing capacity [1]. Both play a critical role in maintaining embryo homeostasis under low-oxygen conditions (hypoxia). Specific processes that take place during embryonic lung development and structures in animals that live at high altitudes are the basis for highly efficient pulmonary ventilation, which leads to altitude adaptation [2], [3]. For plain chickens at low altitudes, inadequate oxygen exchange results in hypoxia syndrome and is lethal [4]–[6]. Therefore, determining the particular altitude adaptation characteristics of high-altitude chickens is important. Improving ventilation and protecting organs is an important strategy against the high death rate in normal animals raised at high altitudes, and studies such as these may suggest therapeutic targets for hypoxia syndrome. However, the molecular mechanisms of chicken lung development and hypoxia adaptation at high altitude have not been well defined.

For *in vitro* incubation, chickens are often used to examine the interaction between embryonic development and environmental conditions. In contrast to mammals, in chicks the mechanisms that underlie the development of their lungs involve a complex morphology and the generation of a cross-current system. Simple lung buds begin to develop at embryonic day (E4), but a complex gas-exchanging lung does not develop until E20–21, when the chick hatches from the shell. Pulmonary ventilation is an important factor for later-stage embryos. Makanya and colleagues have found that during chicken embryo development, the atrium, infundibulate, and air capillaries (ACs) form in turn from E8 to E19. During development, the ACs branch and anastomose with their neighboring cognates during E16–19. This process is a direct result of epithelial cell attenuation and canalization of the ACs [7]. Increasing evidence shows that these processes are accompanied by mesenchyme reduction via apoptosis. Lung mesenchyme degradation brings the ACs and blood capillaries into close proximity with each other [8].

Areas of high altitude, such as in Tibet, are regarded as “restricted areas of life”. The Tibetan chicken, an indigenous chicken bred at high altitudes, is well known for its high hatchability under conditions of reduced oxygen. The hatchability of lowland breeds is approximately zero under the hypoxia that is simulated at an altitude of ≥4000 m, whereas the natural hatchability of Tibetan chicks can reach 70–80% [4]–[6]. An oxygen-deprived environment limits embryonic development and leads to organ paramorphia [9]. Hypoxia stress adaptation is a characteristic of high-altitude animals. But lowland animals also show low-oxygen–induced adaptation during embryonic development. Maina and Kalenga reported that in rat lungs under hypoxic conditions, the mean thickness of the BGB is half or less than of its thickness under normal conditions [10], [11]. Non-homogeneous effects of hypoxia in internal organs have been observed in avian and mammalian species during development [12], [13]. Specific weights (organ (lung) weight/body weight) are obviously decreased in size from E19 in plain chickens under hypoxia, whereas other organs tend to increase in weight [13]. Thus, hypoxia induces a lower density lung structure. However, the mechanisms of hypoxia-induced embryonic lung development and function remain unclear.

MicroRNAs (miRNAs) are non-coding RNA molecules of 20–24 nucleotides long that regulate the expression of other genes by inhibiting translation or cleaving complementary target mRNAs [14], [15]. miRNA functions include the regulation of cell proliferation, differentiation, apoptosis, maintenance of stem cell pluripotency, tumor genesis, and others [16]–[20]. Several miRNAs regulate proapoptotic and antiapoptotic genes. miR-15a and miR-16, two members of the miR-15a/16 cluster, play a role in proapoptosis regulation by inhibiting the translation of the antiapoptotic protein Bcl-2 via binding to the 3′-untranslated region (3′-UTR) of *bcl-2* mRNA [21]. These two miRNAs do not, however, always work in the same way [22]. Recent reports have focused on the influence of miRNAs on homeostasis and, in particular, on the repression of certain genes during hypoxia [23]. Because miRNAs have the ability to modify gene expression rapidly and reversibly, they are ideal mediators for sensing and responding to hypoxic stress. Their ability to modify regulatory pathways can affect the ability of an organism to survive under and adapt to hypoxic conditions [23]. Therefore, during embryonic development, miRNA-mediated regulatory circuits may provide flexible and conditional alternatives to embryonic development. In this study, we identified the influence of long-term hypoxia stress on lung development and showed that the miR-15a, downregulation of HIF-1, was responsive to the oxygen concentration and induced mesenchymal ablation through direct inhibition of the antiapoptotic gene chicken *bcl-2* by binding to a unique target region. The chick embryo dying may start from an imbalance in homeostasis that begins in the lung. The inhibition of miR-15a or activation of HIF-1 or Bcl-2 may prevent hypoxia-induced lung damage and reduce chick embryo death.

## Materials and Methods

### Ethics statement

All animal work was conducted according to the guidelines for the care and use of experimental animals established by the Ministry of Science and Technology of the People's Republic of China (Approval number: 2006-398). The chicken incubation and chicken tissue samples collection procedure were approved by the Animal Welfare Committee of China Agricultural University (Permit Number: XK622). All efforts were made to minimize suffering.

### Animals

The White Leghorn chickens eggs were obtained from the Chicken Farm of China Agriculture University. The Tibet chicken breed was obtained from Nyingchi Tibet and feeding in the Chicken Farm of China Agriculture University. White leghorn chicken eggs and Tibet (hT) chickens were divided into four groups: hypoxic White Leghorn (hW) chickens and normal White Leghorn (nW) chickens, hypoxic Tibet (hT) chickens and normal Tibet (nT) chickens. The hT group and hW group were incubated under plateau-simulating conditions (37°C; 14% O_2_; that is the similar condition at the 3200 m AMSL (Above Mean Sea Level)). The other two groups (nT, nW) were incubated under normal condition as control (37°C; 21% O_2_). Because of the different hatchability, the starting incubation amount of eggs for each group was with big difference. We used 300, 550, 270 and 300 eggs for nW, hW, nT and hT samples incubation. Finally, the amount of nW, hW, nT, hT samples (eggs/chicks) were 209, 163, 193 and 192, respectively. The different organ samples (brain, lung, heart and liver) were all from these eggs/chicks. We want to make the best value for our egg/chick samples.

### Northern blot analysis

Total RNA was isolated from different tissues from adult chickens and chicken embryos using TRIzol (Invitrogen, Carlsbad, CA, USA). Total RNA (20 µg) was fractionated using a 15% denaturing polyacrylamide/8M urea gel. The RNA was then transferred to a GeneScreen Plus membrane (Perkin–Elmer, Waltham, Massachusetts, USA). The 5′ ends of the DNA probes were labeled with [^32^P]ATP (Amersham, Pittsburgh, PA, USA) using T4 polynucleotide kinase (New England BioLabs, Ipswich, Massachusetts, USA). Hybridization and washing were performed as described [24]. Hybridization signals were detected using a Phosphor Screen (Molecular Dynamics, Sunnyvale, CA, USA).

### RNA purification and real-time PCR

Total RNA was isolated with Trizol reagent (Invitrogen) according to the manufacturer's protocol. After treatment with DNase I (RNase-free; TAKARA), oligo(dT) was used to synthesize single-stranded cDNA from 2 µg total RNA using M-MLV reverse transcriptase (Invitrogen). miRNA-specific reverse transcription (RT) stem-loop primers were as follows:

5sRT: 5′-CTCAACTGGTGTCGTGGAGTCGGCAATTCAGTTGAGAAGCCTAC-3′;

16RT: 5′-CTCAACTGGTGTCGTGGAGTCGGCAATTCAGTTGAGCACCAATA -3′;

15aRT: 5′-CTCAACTGGTGTCGTGGAGTCGGCAATTCAGTTGAGACAAACC-3′;

144R: 5′-CTCAACTGGTGTCGTGGAGTCGGCAATTCAGTTGAGGAGTACA-3′.

miRNA was amplified using SYBR Green (Applied Biosystems, Carlsbad, CA, US) and the TaqMan Universal PCR Master Mix Kit (Applied Biosystems) with the 7900HT Fast Real-Time PCR System (Applied Biosystems). Primers and the TaqMan probe were designed using Primer Select software (Primer Express v2.0, Applied Biosystems). Real-time primer sequences were as follows:

GAPDH forward primer: 5′-CGATCTGAACTACATGGTTTACATGTT-3′;

GAPDH reverse primer: 5′-CCCGTTCTCAGCCTTGACA-3′;

*bcl-2* forward primer: 5′-AGCGTCAACCGGGAGATGT-3′;

*bcl-2* reverse primer: 5′-GCATCCCATCCTCCGTTGT-3′;

HIF-1 forward primer: 5′-CAGGTACAA GAGCAACCAA CCA-3′;

HIF-1 reverse primer: 5′-TGGATAAT GACATGGCTA ATGAATTC-3′

HIF-1 probe: 5′-FAM-AGTTCACCTGAGCCC-MGB-3′;

universal reverse primer: 5′-CTCAACTGGTGTCGTGGAGTC-3′

miR-144: 5′-CGCGGCTACAGTATAGATGATG-3′

miR-15a: 5′-GCTGG TAGCAGCACATAATGG-3′

miR-16: 5′- GCGGAGTAGCAGCACGTAAA-3′

RN5S: 5′- GCTCTGGAATACCGGGTGCTGT-3′ (Table S1 and S2).

### Western blot analysis

Equal amounts of protein were resolved by SDS-PAGE and transferred to polyvinylidene difluoride membranes. Membranes were incubated with Bcl-2 (C-2) antibody (1∶500; Santa Cruz Biotechnology, Santa Cruz, CA, US) or HIF-1α (C-19) antibody (1∶500; Santa Cruz Biotechnology). Primary antibody binding was visualized with horseradish peroxidase–conjugated secondary antibody and detected with enhanced chemiluminescence (Beyotime, Jangsu, China). Secondary antibodies were goat anti–mouse IgG (H+L) (1∶10000; Santa Cruz Biotechnology) and rabbit anti–goat IgG (1∶10000; Santa Cruz Biotechnology). For a loading control, membranes were reprobed with primary antibody against actin (2Q1055; 1∶5000; Santa Cruz Biotechnology).

### Immunohistochemical staining

Paraffin-embedded tissues were sectioned (4 µm thick) and mounted on glass slides. They were then dewaxed followed by antigen retrieval in 0.01M sodium citrate at 95°C for 10 min. Immunohistochemistry was done using mouse monoclonal anti–Bcl-2 (C-2) (IgG1; 1∶100; Santa Cruz Biotechnology) in blocking reagent at 25°C for 20 min. The primary antibody was omitted for negative controls. The slides were then treated consecutively with horseradish peroxidase–conjugated goat anti–mouse IgG (1∶200; Chemicon International, Inc., Temecula, CA, USA) and incubated for 1 h at room temperature. Slides were then incubated using a DAB substrate kit (Roche Applied Science, Indianapolis city, IN, USA), and the color reaction was allowed to develop for 5–10 min. After washing, slides were coverslipped.

### In situ hybridization

In situ hybridization was performed as described [25]. Hybridization was done using the 5′-digoxigenin–labeled miRCURY LNA microRNA Detection Probes anti-gga-miR-16 and anti-gga-miR-15a (EXIQON, Vedbaek, Denmark). The 5′-digoxigenin–labeled Scramble-miR Probe (EXIQON) was used as a negative control.

### TUNEL staining

TUNEL staining was performed using the in situ Colorimetric TUNEL Apoptosis Assay Kit (Beyotime), according to the manufacturer's manual. Briefly, incubated the frozen sections in the cold 0.1% Triton X-100 for 2 min. Then moved the slides into the 0.3% H_2_O_2_ in methanol for 2 min at RT. Washed 3 times with PBS. That was followed by incubation with TdT enzyme solution for 60 min at 37°C. The reaction was terminated by incubation in a stop/wash buffer for 10 min at 37°C. Then used the Streptavidin-HRP for coloration. Incubated the sections in the Streptavidin-HRP working solution for 30 min at RT, following with the colour reagent incubation for 30 min.

### Cell culture

The human embryonic kidney 293T cells (American Type Culture Collection, Rockville, MD) were cultured at 37°C in 5% CO_2_ in DMEM supplemented with 10% fetal bovine serum (HyClone, Logan, UT, US) and antibiotics.

### Plasmid transfection and luciferase assay

The chicken *bcl-2* 3′-UTR containing a miR-15a–binding site and a miR-16–binding site was cloned into the psiCHICK-2 vector (Promega, Madison, WI, US) using the following primers:

forward primer: 5′-CTCGAGAGTCACCCAGTTTATCGT-3′;

reverse primer: 5′-CTCGAGGATTCTTCCGCTTCGTCA-3′.(Table S3)

The synthesized fragments containing the reverse complement sequences of gga-miR-15a and gga-miR-16 were also individually cloned into the psiCHICK-2 vector and used as a positive control. The psiCHICK-2 vector was transfected into the human embryonic kidney 293T cells with miR-15a/16 mimics (Applied Biosystems) or a miR-15a/16 inhibitor (Applied Biosystems) or a negative control (Applied Biosystems). The concentration of mimic is 10 pmol. The relative proportion of mimic and inhibitor was 1∶10. The firefly and Renilla luciferase activity of each transfection was determined with the Dual-Glo Luciferase Assay System (Promega) 44 h post-transfection.

## Results

### The expression of miR-15a and miR-144 is induced by hypoxia stress in chicken embryo

**Figure 1:**
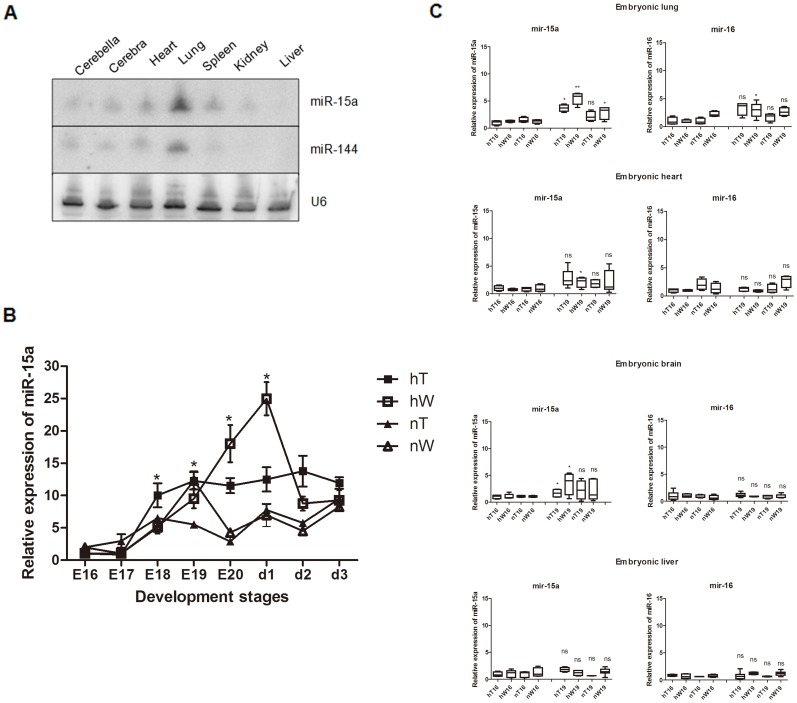
Expression of miRNAs in chicken lung tissues. (A) Total RNA from tissues from E19 chicken embryos was blotted with probes for miR-15a, miR-144 and U6 (loading control). miR-15a and miR-144 were identified in late-stage embryonic lung tissue. In each sample, the total RNA was mixed with samples from 9 chickens. (B) Quantitative expression analysis of miR-15a showed hypoxia-related expression that was affected by both the species and environmental conditions. The hW chicken group was most sensitive relative to other three groups. In the nW chicken group, there was a response at E19. At E19 of the nW chicken group, the expression of miR-15a remained relatively high in the hT chicken group compared with the nT, nW chicken groups and was largely unchanged in the nT chicken group through the whole embryo stages. Data are expressed as the mean ± SEM for each group. (C) Quantitative expression analysis of miR-15a and miR-16 in the embryonic lung, heart, brain and liver at E16 and E19 tissues and were expressed as the mean ± SEM for each group. Under hypoxia stress, miR-15a was more highly expressed at E19 than at E16 in the brain, heart and lung for the hW group and in the lung and brain for the hT group. miR-16 showed a weak response to stress in the embryonic lung (hW). Result statistically different are indicated with an asterisk/s (* *P*<0.05; ** P<0.01; ns : not significant). E16-20  =  embryonic d13-20, respectively. d1, d2, d3  =  the 1^st^ day after hatching, the 2^nd^ day after hatching, the 3^rd^ day after hatching.

Xu and colleagues [26] published that the expression levels of both miR-15a and miR-144 are higher in the lung tissue. Our studies confirmed the result and the tissue special expression. In later stages of chick embryo development, relatively strong expression of miR-15a and miR-144 was detected in lung tissues with Northern blotting, whereas expression was weak in other organs (Fig. 1A). To determine whether both miR-15a and miR-144 are highly expressed throughout chicken embryonic development or just a temporally variable expression pattern, we compared the miR-15a and miR-144 expression profiles in lung tissues. We set the hatcher to simulate the plateau condition (AMSL is 3200 m, 37°C, 14% O_2_, PO_2_ is 14.093 kPa) and incubated both the Tibet chicken eggs and White Leghorn chicken eggs in it to simulate the hT and hW groups. The nT and nW chicken groups are the eggs incubated in normal plain condition (37°C, 21%O_2_, PO_2_ is 100 kPa). Lung samples were obtained from E13 to the third day after hatching (d3). Real-time PCR showed no difference in miR-15a and miR-144 expression from E13 to E17 in the four groups (data not shown). At E18, the hT group showed significant upregulation of miR-15a that remained relatively high and stable through d3 (Fig. 1B). miR-15a in hW showed continuously increasing expression from E18 and a peak at E20 and the hatch out day (d1) (Fig. 1B). Expression of miR-15a in nW chickens nearly coincided with that of nT chickens except for a relative peak value for nW at E19 (Fig. 1B). As a control, miR-15a in nT chickens showed a relatively smooth line, indicating stable, low expression of miR-15a (Fig. 1B). A comparison of interclass differences showed that at E18, expression of miR-15a was higher in lung tissues from hT than in the lung tissues from the other three groups. At E19, the time at which the respiratory CCGS develops, miR-15a was also upregulated in hW and nT chickens. Comparing the expression levels at E20, we found that expression of miR-15a was relatively low in both tissues under normal conditions (nT, nW chickens), and relatively high in the hW and hT sample which incubated under plateau simulating condition (Fig. 1B). Quantitative analysis of miR-144 revealed no significant changes over time (data not shown). As a cluster and family member, miR-16 was reported to have the same function as miR-15a [21]. To determine whether the peak value of miR-15a expression was lung specific or was a systemic reaction to hypoxia and whether expression of miR-15a was the same as that of miR-16, we performed real-time PCR for miR-15a and miR-16 using chick embryo lung, heart, brain and liver at E16 and E19. hW showed remarkable upregulation of miR-15a at E19 as compared with that at E16 in the embryonic lung (Fig. 1C). In the heart and brain, miR-15a expression in hW and hT was also increased from E16 to E19, but the range was not as large as that in the lung (Fig. 1C). In the liver, no differences in expression between the two embryonic stages or among the experimental groups were identified (Fig. 1C). We identified a small increase in miR-16 from E16 to E19 in embryonic lung in hW (Fig. 1C). However, there were no clear changes in miR-16 in the other groups. Thus, miR-16 also appeared to be affected by hypoxia at a time when the respiratory gas exchange was developing. Thus, miR-15a expression was sensitive to oxygen concentration, and the strongest response was detected in lung tissue. We also found differences in hypoxia-induced expression of miR-15a and miR-16 between high-altitude and plain chicks. The period of development of pulmonary ventilation is crucial for development of lung organ function. Although miR-16 belongs to the same cluster and family as miR-15a [21], they were differentially sensitive to hypoxia. miR-144 also appeared responsive to hypoxia (data not shown), but it is not involved in temporal lung development.

### Hypoxia stress upregulates HIF-1α and Bcl-2 protein

**Figure 2:**
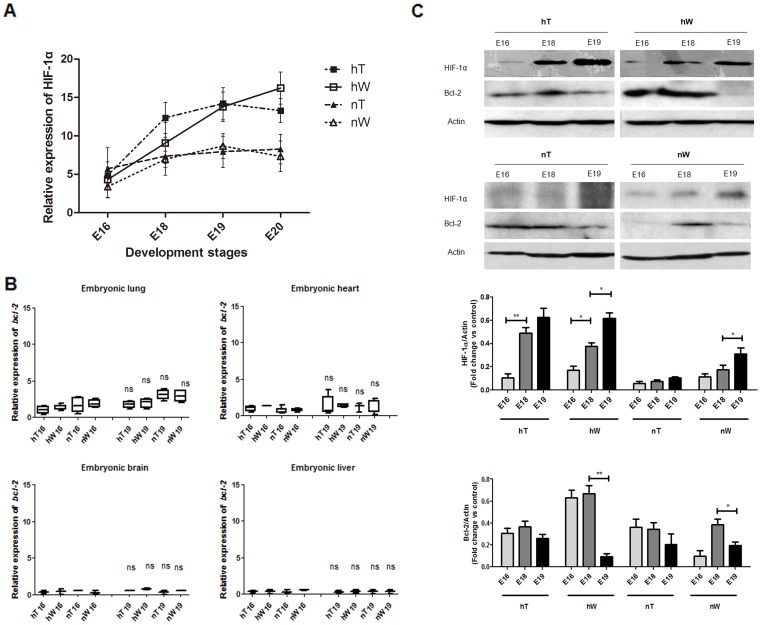
HIF-1α and Bcl-2 expression in lung tissue under hypoxia and normoxia conditions. Data were obtained from hT, hW, nT, and nW chicken tissues at E16, E18, E19, and E20. (A) Quantitative expression analysis of HIF-1α mRNA in embryonic lung at E16, E18, E19, and E20. The stress reaction in hW was robust as compared with the smooth response in hT. Data are expressed as the mean ± SEM for each group. (B) Quantitative expression analysis of *bcl-2* mRNA in the embryonic lung, heart, brain and liver at E16 and E19. There were no changes in the expression of *bcl-2* mRNA between the E16 and E19 in each kind of embryonic tissues. Data are expressed as the mean ± SEM for each group. (C) Analysis of HIF-1α and Bcl-2 expression at the protein level in lung tissue (shown in western blot and densitometry value). HIF-1α protein expression increased from E16 to E19, however in different level in hT, hW, nT, and nW group. Bcl-2 protein did show different levels of expression across different time points and different groups. (* *P*<0.05; ** P<0.01; ns  =  not significant).

Hypoxia-inducible transcription factor 1 alpha (HIF-1α) is either anti-apoptotic or pro-apoptotic, according to the cell type and experimental design. Because severe or prolonged hypoxia induces apoptosis [27], HIF-1 can be used to monitor this hypoxia-induced response. To explore the reaction of the lung to hypoxia, we detected HIF-1α transcripts using quantitative PCR and HIF-1α protein using western blotting (Fig. 2A, C). Hypoxia-induced HIF-1α mRNA expression in hW increased from E16 to E19, whereas HIF-1α in hT kept higher (from E18 to E19), until E20 the trend reverse. In the normoxia experimental groups, nW and nT chickens, HIF-1α mRNA expression showed a slight upward trend, but there was no difference between these two groups (Fig. 2A). These changes were further confirmed with Western blotting, which showed that HIF-1α protein was induced by hypoxia stress (Fig. 2C). Interestingly, HIF-1α protein in nW chickens was upregulated compared to nT chickens, especially at E19 (Fig. 2C). During normoxia, HIF-1α expression can be regulated at the translational level [28]. These results suggest that nW also suffered acute hypoxia stress at E19.

Bcl-2 is downstream of HIF-1α in the HIF-1α–induced anti-apoptotic pathway [29]. Because hypoxic cell death is prevented by anti-apoptotic proteins such as Bcl-2 [30], we investigated *bcl-2* mRNA expression during the whole embryo development and identified no remarkable variation in *bcl-2* mRNA (Fig. 2B, and data not shown). However, Bcl-2 protein was strongly decreased in E19 hW lung samples as compared with its expression at E16 and E18 (Fig. 2C). Moreover, in other groups, Bcl-2 protein levels were decreased to different degrees in association with the degree of hypoxic stress (the time length) (Fig. 2C). Thus, the regulation of Bcl-2 protein levels may not be at the transcriptional level but rather at the translational level. We note that miR-15a expression was increased at E19 during hypoxia (Fig. 1B).

### *bcl-2* is a target gene of miR-15a but not miR-16 in chicken lung tissues under hypoxia

**Figure 3:**
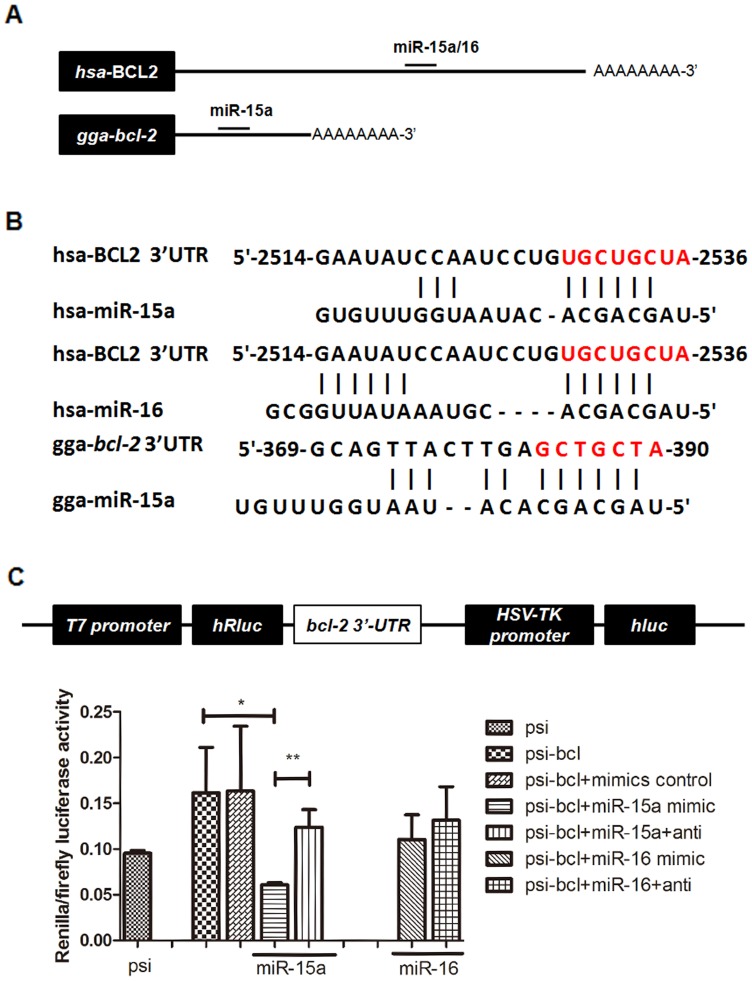
*bcl-2* is a target gene of miR-15a, but not miR-16 in chicken lung. (A) miR-15a regulates the translation of *bcl-2* mRNA through a sequence that is not conserved with human sequence. miR-15a and the binding site in the gga-*bcl-2* 3′-UTR are shown, but miR-16 shows no target site in this part of the sequence. (B) miR-15a/16 have a consistent target site in human *bcl-2* 3′-UTR. The miR-15a binding site in the *bcl-2* 3′-UTR sequence mediates translation repression by miR-15a. (C) The luciferase reporter vector contains two luciferase cDNAs, Renilla luciferase (hRluc) and firefly luciferase (hluc). The *bcl-2* 3′-UTR was fused to the hRluc cDNA downstream sequence. In co-transfected cells, the miR-15a mimic decreased the expression of hRluc and miR-15a mimic inhibitor rescued hRluc activity; no differences were seen for miR-16. Data are expressed as the mean ± SEM. * *P*<0.05, ** *P*<0.01.

miR-15a and miR-16 negatively regulate *bcl-2* by directly binding to a particular sequence in the 3′-UTR of *bcl-2* and inhibiting its translation [21], [31]. This repression is sufficient to induce apoptosis [32]. Our observation of hypoxia-induced miR-15a expression (Fig. 1B) and reduced Bcl-2 protein levels at E19 (Fig. 2C) indicated an inverse relationship between miR-15a and Bcl-2 protein expression, which suggested a causative role for miR-15a in the downregulation of *bcl-2*. However, as a cluster member of miR-15a, miR-16 was identified as hypoxia insensitive. To further verify whether miR-15a or miR-16 repressed chicken *bcl-2* by binding directly to the predicted binding site in its 3′-UTR as shown in human cells [31], we analyzed the complementation of the chicken *bcl-2* mRNA (NM_205339.1) and chicken miR-15a/16 using four different prediction algorithms: TargetScan [33], RNA22 [34], DIANA [35], [36], and PicTar [37]. Interestingly, human *bcl-2* mRNA (NM_000633) has one target located at nt 2529–2536 of its 3′-UTR [21] that is not conserved with the chicken bcl-2 3′-UTR. Chicken bcl-2 was predicted to contain a miR-15a/16: bcl-2 site located at nt 369–390 of its 3′-UTR (Fig. 3A). The RNA22/PicTar results showed that the target site sequence complemented only with chicken miR-15a, and the TargetScan result suggested that both the chicken miR-15a and miR-16 would work (data not shown). We cloned the entire chicken *bcl-2* 3′-UTR, containing the miR-15a/16 binding site, and inserted it into the multiple cloning sites of the psiCHICK-2 vector. We transfected this luciferase reporter vector into 293T cells. We also co-transfected these cells with chicken miR-15a mimic, mimic and inhibitor, or mimic control (miRNA mimics are small, chemically modified double-stranded RNAs that mimic endogenous miRNAs and enable miRNA functional analysis by up-regulation of miRNA activity. miRNA inhibitors are small, chemically modified single-stranded RNA molecules designed to specifically bind to and inhibit endogenous miRNA molecules and enable miRNA functional analysis by down-regulation of miRNA activity. The controls are small control molecules). We performed the same experiment with chicken miR-16. Chicken miR-15a decreased luciferase activity of the reporter vector containing the 3′-UTR sequence, and the level of luciferase activity recovered in the presence of the chicken miR-15a inhibitor. However, no change was observed with the chicken miR-16 mimic (Fig. 3C). Together, the results suggested that chicken miR-15a decreased chicken *bcl-2* translation by directly acting on a miR-15a–specific response element in the 3′-UTR of chicken *bcl-2* mRNA and that this effect may be different in chicken *bcl-2* as compared with that in human *bcl-2*. Chicken miR-16, another member of the cluster, did not affect *bcl-2* regulation.

### Hypoxia-induced miR-15a overexpression promotes the formation of CCGS and a thin BGB

At E19, the lung begins to function, but the development of the complex CCGS is still occurring. To further understand a possible role for hypoxia-induced miR-15a in the development of a functional lung in vivo, we immunostained sections from hT, hW, nT, and nW chicken lungs for Bcl-2 protein and performed terminal deoxynucleotidyl transferase nick-end labeling (TUNEL) staining.

**Figure 4:**
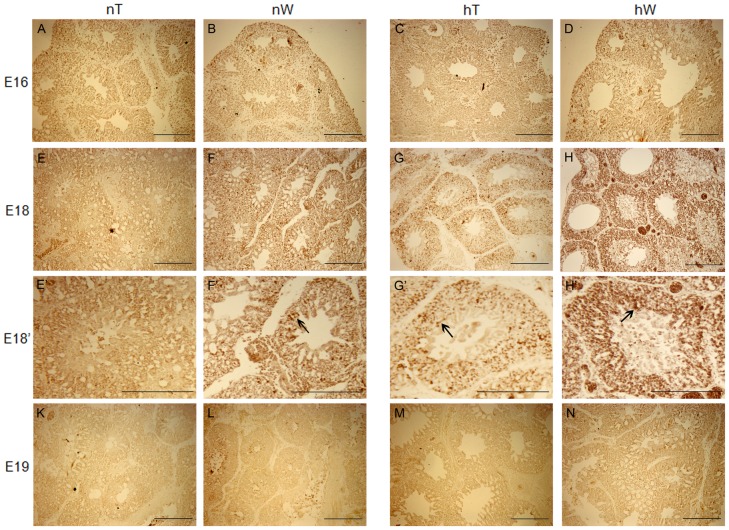
Immunohistochemical staining (dark brown coloring) for Bcl-2 protein in lung specimens from chickens incubated under normal and hypoxic conditions. Bcl-2 protein expression in lung samples from nW, hT, and hW gradually increased moderately from E16 to E18 before the onset of lung functioning (E–H), and disappeared at E19. By E18 in nW lungs, weaker Bcl-2 expression was observed relative to hT (G) and hW (H) chicken groups. In nT chicken sections, Bcl-2 protein staining was nearly unchanged throughout the developmental stages examined (A, E, K). To see details of the E18 lung structure, higher-magnification photomicrographs were taken. Bcl-2 staining was detected in the mesenchyme (arrows) around the ACs and not in the infundibula or atrias of chicken lung (F’, G’, H’)”. In the hW section at E18, Bcl-2 staining was strong (arrow in H’), but weaker in hT and nW at this stage (arrows in G’, F’). Scale bar  =  200 µm.

Hypoxia upregulated Bcl-2 protein expression at E18 (Fig. 4G, H). The strongest staining was of hW chicken lung sections at E18 (Fig. 4H). Staining of hT and nW lung sections at E18 were similar. Their staining were weaker than what detected of the hW sections (Fig. 4F, G). No staining was observed in nT chickens at E18 (Fig. 4E). As expected, the staining was localized in the mesenchyma around the ACs of the chicken lung (Fig. 4F’, G’, H’). No special staining signal was detected in other sections (Fig. 4A, B, C, D).

**Figure 5:**
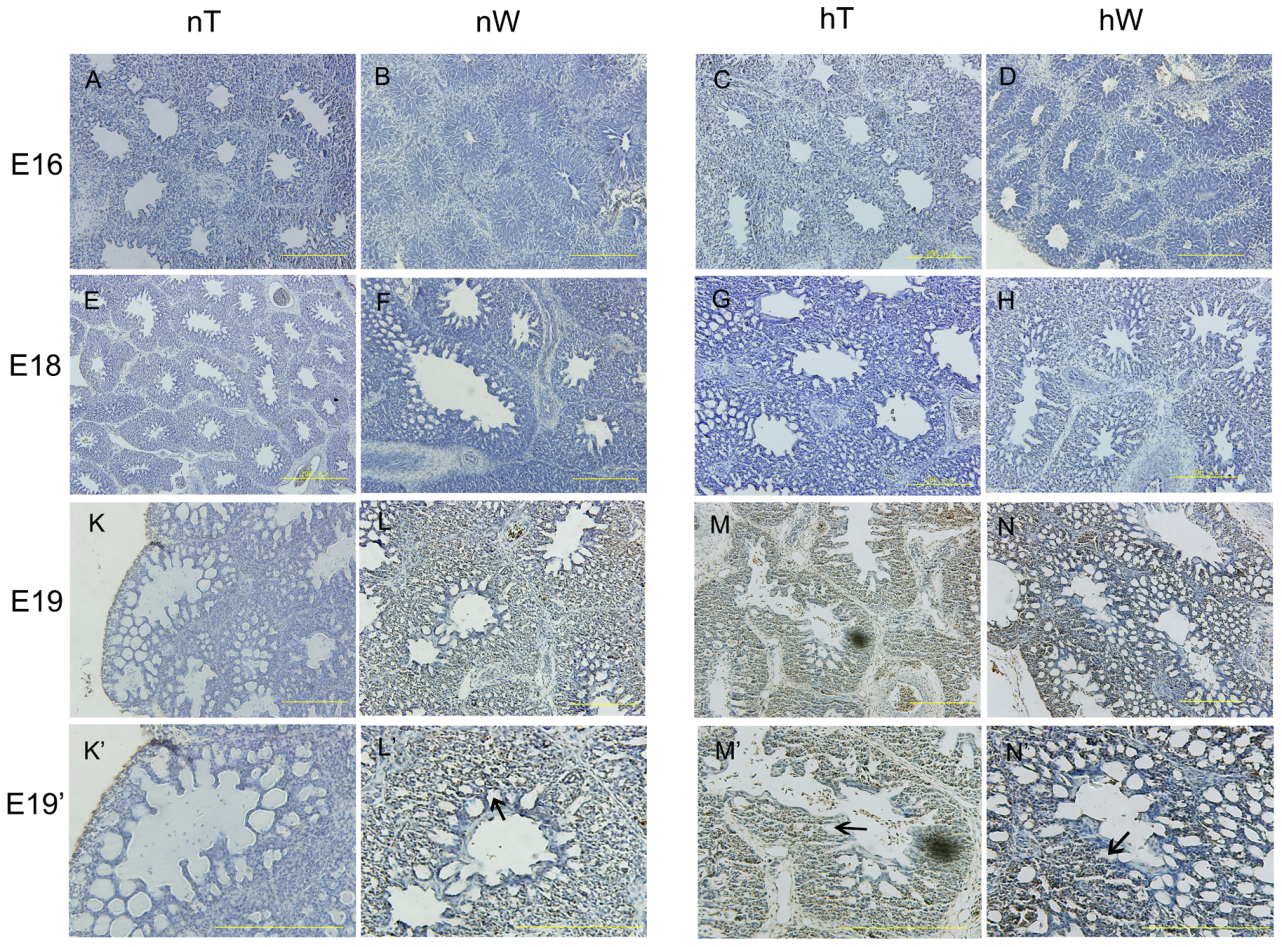
Analysis of apoptosis in lung specimens from chickens incubated under normal and hypoxic conditions. From E16 to E18, no TUNEL staining was identified (A–H). At E19, apoptotic cells were localized in the mesenchyme surrounding the atrias and infundibula of the chicken lungs (L, M, N). There was no obvious staining in nT chicken at E19 (K). At higher magnification, staining was clearly seen in the regions between ACs and not in the parabronchi, atrias, or infundibula (arrows in L’, M’, N’). hW staining at E19 (arrow in N’) was clearly darker than that observed in hT or nW lung sections at this stage (arrows in M’, N’). The tube density in hW at E19 was also higher than that in sections from those other two groups. Scale bar  =  200 µm.

Apoptotic cells were observed at E19, except for in nT chickens (Fig. 5K). TUNEL staining was darker in hW lung sections at E19 (Fig. 5N) and in hT lung sections at E19 (Fig. 5M) as compared with staining in nW lung sections at E19 (Fig. 5L). Sections viewed at higher magnification showed that apoptotic cells were located in the mesenchyme around the migrating epithelial tubes, including capillaries (Fig. 5L’, M’, N’). Interesting, at E19, no significant difference of Bcl-2 protein expression was detected in all the groups (Fig. 4K–N). Taken together, these results suggested that the lung appeared resistant to apoptosis at E18, which is likely the result of upregulation of Bcl-2. However, at E19, the time of further lung development, the tissues became more sensitive to the oxygen concentration, and apoptosis was induced by the hypoxia-induced upregulation of miR-15a. Of interest, the tube density in hW at E19 was also higher than that in sections from those other two groups. That indicated adaptive lung development which has also been reported in mammals living as altitude adaptation [3], [38]. These responses to hypoxia stress indicate an oxygen-concentration dose effect process in the chick lung. The apoptotic cells were located in the mesenchyme around the migrating tubes. These mesenchymal cells are in direct contact with the air and are the targets of apoptosis, which may lead to expansion and migration of tubes, implying precise temporospatial regulation. During the subsequent CCGS establishment when the ACs are branching and anastomosing with their neighboring cognates, mesenchymal cell apoptosis participates in the approximation of ACs and blood capillaries (BCs), to establish a thin BGB.

## Discussion

Adaptation to hypoxia of the Tibet chicken appears to involve numerous changes at the organism's level. These chicken have bigger hearts and lungs, higher hemoglobin oxygen transfer efficiency and other adaptation characteristics [13], [39]–[42]. Such adaptive changes may counteract the reduced oxygen uptake and mitigate tissue injury due to hypoxia. There are two oxygen sensitive periods during the chick embryo development, the embryo day 0–4 (early stage) and the respiratory gas exchange (around the E19, the late stage) [43]. Increased oxygen tension in the early and late stages of development results in increased hatchability, while during while the middle stage of development increased oxygen causes little or no change [44]–[46].

**Figure 6:**
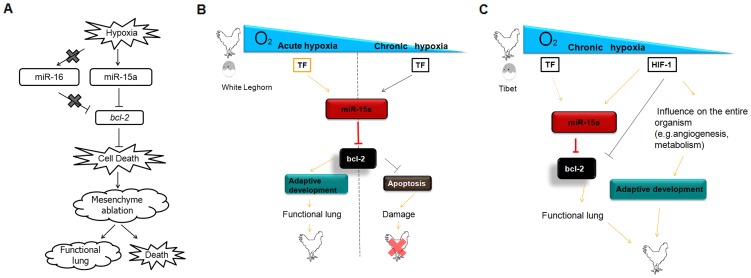
Schematic representation of hypoxia-induced gga-miR-15a control of mesenchymal cell death/ablation during functional lung establishment and adaptive development. (A) In chicken lung, hypoxia stress stimulates gga-miR-15a expression, and miR-15a may bind to the 3′-UTR of gga-*bcl-2* and inhibit its antiapoptotic activity through posttranscriptional gene silencing. Mesenchymal apoptosis then occurs, which leads to a functional lung with a thinner BGB or an acute reaction resulting in death in different chicken species. gga-miR-16 shows no response to hypoxia, and there is no target site for gga-miR-16 in the 3′-UTR region of gga-*bcl-2*. (B) Mesenchymal cell death/ablation indeed appeared necessary for the formation of a thinner BGB and is promoted by hypoxia (the oxygen concentration). Particular transcriptional factors (TF) may respond to the hypoxia stress and induce gga-miR-15a expression. For plain chickens, the induced apoptosis would be helpful for the formation of a thinner BGB, but it could be lethal if they are exposed to low oxygen concentrations for a long time. (C) However, for high-altitude chickens, apoptosis is genetically initiated to form a functional BGB, and it is under control of the whole organism (C). Acute hypoxia: related hypoxia condition induced by the CCGS occursing around E19. Chronic hypoxia: long-term altitude condition with low oxygen concentration.

The major finding of our study is that, during the late stage of chick embryo development, lung mesenchymal cells become sensitive to the oxygen concentration when the lung assumes the gas-exchange function from the allantois at E19. Hypoxia stress induces proapoptotic chicken miR-15a expression and subsequently inhibits the translation of the antiapoptotic protein Bcl-2, resulting in mesenchyme ablation. Chicken miR-16, a cluster and family member of chicken miR-15a, does not affect the hypoxia-induced pathway. In vivo, mesenchymal cell death was seen around the tubes, and thus, apoptosis led to expansion and migration of new tubes. The mesenchyme ablation led to the establishment of CCGS and a thin BGB. Thus, BGB formation was associated with the oxygen concentration. The distance between ACs and BCs will also be affected by this process. Because differences exist among species, long-term hypoxia stress in normal chicks is lethal. The lung is the most sensitive organ at later incubation times, and an imbalance between organ ablation and body development will be lethal (Fig. 6A). To our knowledge, our experiments have provided the first evidence that establishment of a thin BGB can be mediated by the oxygen concentration during the sensitive period of lung development. Development of a thin BGB requires precise temporospatial regulation and is tightly correlated with the incubation conditions. The response to stress controls mesenchymal cell death via a miRNA-mediated mechanism. We also show that of the two cluster and family members, only chicken miR-15a is responsive to hypoxia stress and participates in downstream regulation.

The ability to sense and respond to hypoxia is of fundamental importance during embryonic development as well as environmental adaption. Dysregulation of oxygen homeostasis is an important cause of embryo death in hypoxic conditions. An organism's oxygen homeostasis begins with the lung gas-exchange system. Among the extant vertebrate taxa, the avian lung is reported to be the most efficient and complex gas exchanger and is a CCGS [47], [48]. Microscopy was used to identify the functional development of the avian lung in 2006 and showed establishment of a thin BGB by the time of hatching [8], [49]–[52]. However, the lung of various domestic chicks begins to develop at E18 [8]. On later days (around E19), the mesenchymal cells of chick lung immediately surrounding the migrating epithelial tubes become the initial targets of apoptosis. In this study, we report for the first time that at E19, initiation of pulmonary ventilation, which brings air into the lung ACs, was induced immediately and promoted establishment of an oxygen concentration–dependent CCGS. The induced establishment of the CCGS was implemented by induced mesenchymal apoptosis, resulting in expansion and migration of tubes. The observation that the lung organ/body weight ratio of normal chickens was lower than in hypoxic conditions indirectly reflects the hypoxia-induced disintegration of mesenchymal cells [13]. Adaptive lung development has also been reported in mammals living at higher altitudes [38]. E19 is the peak of mortality for embryos growing in both normal and hypoxic conditions [53]. Our data showed relative hypoxic stress at E19 even when embryos are incubated in normal conditions. We suggest that when an acute injury disrupts developmental homeostatic regulation, hypoxia may result in the of the affected animal.

miRNAs were discovered nearly two decades ago, and their importance was highlighted when they were identified as conserved in higher eukaryotes. Recent reports have focused on the influence of miRNAs on homeostatic and adaptive development, and in particular, response to hypoxia. An ideal mediator of hypoxic stress should be able to modify gene expression both rapidly and reversibly, a widely accepted characteristic of miRNAs [23]. Therefore, miRNAs may be a molecular rheostat that facilitates survival and adaptation to hypoxic conditions by tuning and switching regulatory circuits on and off. Various miRNAs appear to regulate hypoxia responses in a variety of different organisms, cell types, and disease states [22], [54]–[57]. More than 90 hypoxia-regulated miRNAs (HRMs) have been described, and many regulate certain cell types only. However, as a general characteristic, these HRMs respond to hypoxic stress rapidly (less than 48 h). These hypoxic stress responses are regulated through different pathways. miR-107 is directly regulated by p53, which is one of the most frequently mutated genes in human cancers [58]–[60]. HIF-1 was identified as target gene of miR-107 both in vitro and in vivo, suggesting a p53-miR-107-HIF-1 hypoxia response pathway [60]. miR-206 was also proved as a direct regulator of HIF-1alpha. In cultured hypoxic pulmonary artery smooth muscle cells (PASMCs), miR-206 promotes the Hypoxia-induced pulmonary hypertension though the HIF-1a/Fhl-1 pathway [61]. HIF-1 and microRNAs build a network of hypoxia response, HIF-1 was reported as regulator of miR-9, it induced upregulation of miR-9 in PASMCs. As a phenotypic switch, knockdown of miR-9 was followed by hypoxia exposure attenuation in PASMCs proliferation [62]. miR-210 and miR-373 were discovered in HeLa and MCF-7 cells. Both miRNAs contain hypoxia-responsive elements (HREs) in their promoter regions, suggesting direct regulation by HIF-1 [63]. Most hypoxic injury results in cell apoptosis. Hayashita and Frankel reported that miR-21 and the miR-17-92 cluster function as antiapoptotic miRNAs in various cancers [64], [65]. In contrast, Cimmino et al. showed that miR-15a and miR-16 promote apoptosis by posttranscriptional gene silencing of *bcl-2* [21]. In this study, our data suggested that the expression of miR-15a, but not miR-16, is sensitive to the oxygen concentration and was significantly increased in lung mesenchymal cells in chicken. This results in select apoptosis in mesenchymal cells around the migrating tubes through reduction of Bcl-2 levels, as shown in vitro and vivo. Although miR-15a and miR-16 belong to the same cluster and miRNA family, Yin *et al*. also reported that miR-15a plays a causative role in the regulation of apoptosis by directly targeting *bcl-2* during ischemic vascular injury [22]. Together, our experimental data extend these early findings and further show that chicken miR-15a can repress *bcl-2* translation by directly binding to the 3′-UTR of *bcl-2* with a different target site sequence. To our knowledge, our work is the first to document that chicken miR-15a, but not miR-16, regulates apoptosis by directly targeting chicken *bcl-2* at a specific target region during later chick lung CCGS development.

Kang *et al.* [66] reported that the miR-15a/16-1 cluster is a homo-cluster. Because the homologous miRNA in the homo-cluster has almost the same proximal targets or genes in the same pathway, homo-cluster miRNAs show a direct regulatory coordination involving one step. Thus, there is no intermediate regulator between the miRNAs and their targets; direct coordinated regulation has the advantage of accuracy and quickness. Hua and colleagues identified a group of regulatory miRNAs, including miR-16, which regulates the expression of vascular endothelial growth factor [67]. miR-16 may function during BC network formation, which is perpendicular to the long axis of ACs. miR-16 shows unequal expression among tissues [68]. In this study, we suppose that in chick, members of the miR-15a/16-1 cluster respond to hypoxia through different target genes and pathways. We showed relatively high expression of miR-144 in chicken embryo lungs. Analysis of miR-144 showed a lower response to hypoxia. miR-144 expression has been reported to be limited to the blood islands at stage 8 [69], and it is crucial for erythroid homeostasis [70]. miR-144 also modulates oxidative stress tolerance and is associated with anemia severity in sickle cell disease [71]. Thus, we suggest that miR-144 may be responsive to hypoxia but may have no direct relationship with lung development.

Lung organogenesis is a field that has attracted many investigators. Since “molecular embryology” was reviewed, more challenges have emerged in developmental lung biology. miRNAs play an important role not only in cell proliferation and tumorigenesis but also in apoptosis that is associated with organism and organ development. Yang et al. identified 167 differentially expressed miRNAs during rat lung organogenesis, and Dong reported 117 miRNAs that are dynamically and temporally regulated during mouse lung organogenesis. Mujahid et al. found that, during the important window in lung development, 25 miRNAs showed different expression profiles across gestation, and 37 miRNAs changed between males and females [72]–[74]. Transcription factors connect the extracellular environment to the signaling network in cells. HIF-1 is a widely recognized hypoxic stress transcription factor [23]. In a recent report, HIF-1 was shown to function in lung branching morphogenesis and epithelial maturation. Functional loss of HIF-1 results in neonatal lethality and severe respiratory distress. Interestingly, some abnormalities were detected in the endothelial barrier, which indicates a disorder in development of the epithelial and mesenchyme [75]. By controlling both O_2_ delivery and utilization, HIF-1 functions as a important regulator of O_2_ homeostasis. Under tissue hypoxia or ischemia, HIF-1 protect the heart against injury by inducing angiogenic growth factors, which stimulate vascular remodeling to increase blood flow following [76]. Intrestingly, for the High altitude adaptive species, during the Tibet chicken embryo lung development, HIF-1 and other factors induce complex CCGS and a thin BGB which is necessary for the High altitude adaptive lung function. In the present study, we detected upregulation of both HIF-1 and miR-15a with a similar expression profile during E16–E19. We hypothesize that the induced ablation of mesenchymal cells may involve HIF-1 as a hypoxia sensor, which then indirectly decreases Bcl-2 protein levels by inducing miR-15a expression. However, more studies are needed to elucidate the details. Other transcription factors are likely to participate in this regulatory network. The sensor of oxygen concentration is important here, because apoptosis occurs only around the ACs and not at the top to the atria. In the Tibet chicken, induced apoptosis is necessary for establishment of functional lungs containing complex CCGS and a thin BGB. However, when the induced apoptosis is uncontrolled and organ homeostasis is lost, normal chickens will die for pressure overload (Fig. 6B, C). Other molecules have been reported as miR-15a regulators in different cell types or tissues, and some are hypoxia regulated. miR-709 responds to apoptosis stimuli and prevents expression of miR-15a/16 in mouse [77]. Myc and HDAC3 are regarded as miR-15a/16 regulators in mantle cells and other non-Hodgkin B-cell lymphomas [78], [79]. HERΔ16, a clinically important oncogenic isoform of HER2, is another regulator of miR-15a/16 in breast tumors [80]. In human tumors, E2F1 is a positive regulator of miR-15a and miR-16, but only miR-15a inhibits expression of cyclin E [81]. In a mouse model of ischemia-induced cerebral injury, PPARδ (peroxisome proliferator-activated receptorδ) was identified as a regulator of miR-15a that inhibits the induction of apoptosis by preventing miR-15a expression [22]. Intrestingly, three isoforms of PPARs (α,γand δ) were all documented direct downstream of miRs [22], [82], [83]. PPRAα were identified had specific functional variants between highland Tibetan populations and normal [85]. However, although PPRAα is in the Gene Ontology biological process ‘response to hypoxia’, but it is not even in the HIF pathway [84]. So, additional studies will be needed to further understand the interaction between miRNAs, gene functional variation and conditional development.

In this study, we identified the influence of long-term hypoxia stress on lung development. In conclusion, the mechanisms of hypoxia-induced, miR-15a–mediated CCGS and thin BGB establishment are important for understanding adaptational development of chick lung at particular stages. HIF-1 may regulate miR-15a in this setting. We also showed that only miR-15a and not miR-16 was responsive to the oxygen concentration and induced mesenchymal ablation through direct inhibition of the antiapoptotic gene chicken *bcl-2* by binding to a unique target region. The stage at which embryos die may results from an imbalance in homeostasis that begins in the lung. Therefore, either inhibition of miR-15a or activation of HIF-1 or Bcl-2 may potentially be therapeutic options for preventing hypoxia-induced lung damage and may reduce death at particular stages of chick embryo development.

## Supporting Information

Table S1 **Reverse transcription primers and run method.**
(DOCX)

Table S2 **Real-time PCR primers and run method.**
(DOCX)

Table S3 ***bcl-2*** **3′-UTR cloning primers.**
(DOCX)

## References

[1] Powell FL (1982) Diffusion in avian lungs. Fed Proc 41: 2131–2133. 7075785

[2] Tang JR, Seedorf GJ, Muehlethaler V, Walker DL, Markham NE, et al (2010) Moderate postnatal hyperoxia accelerates lung growth and attenuates pulmonary hypertension in infant rats after exposure to intra-amniotic endotoxin. Am J Physiol Lung Cell Mol Physiol 299: L735–748. 20709730 10.1152/ajplung.00153.2010 PMC3006274

[3] Durmowicz AG, Hofmeister S, Kadyraliev TK, Aldashev AA, Stenmark KR (1993) Functional and structural adaptation of the yak pulmonary circulation to residence at high altitude. J Appl Physiol 74: 2276–2285. 8335557 10.1152/jappl.1993.74.5.2276

[4] Visschedijk AH, Ar A, Rahn H, Piiper J (1980) The independent effects of atmospheric pressure and oxygen partial pressure on gas exchange of the chicken embryo. Respir Physiol 39: 33–44. 7361018 10.1016/0034-5687(80)90012-2

[5] Visschedijk AH, Tazawa H, Piiper J (1985) Variability of shell conductance and gas exchange of chicken eggs. Respir Physiol 59: 339–345. 3992066 10.1016/0034-5687(85)90137-9

[6] Visschedijk AH (1985) Gas exchange and hatchability of chicken eggs incubated at simulated high altitude. J Appl Physiol 58: 416–418. 3920187 10.1152/jappl.1985.58.2.416

[7] Makanya AN, El-Darawish Y, Kavoi BM, Djonov V (2011) Spatial and functional relationships between air conduits and blood capillaries in the pulmonary gas exchange tissue of adult and developing chickens. Microsc Res Tech 74: 159–169. 21275004 10.1002/jemt.20887

[8] Maina JN (2004) Morphogenesis of the laminated, tripartite cytoarchitectural design of the blood-gas barrier of the avian lung: a systematic electron microscopic study on the domestic fowl, Gallus gallus variant domesticus. Tissue Cell 36: 129–139. 15041415 10.1016/j.tice.2003.11.002

[9] Tuder RM, Yun JH, Bhunia A, Fijalkowska I (2007) Hypoxia and chronic lung disease. J Mol Med (Berl) 85: 1317–1324. 18040654 10.1007/s00109-007-0280-4

[10] Maina JN, King AS, Settle G (1989) An allometric study of pulmonary morphometric parameters in birds, with mammalian comparisons. Philos Trans R Soc Lond B Biol Sci 326: 1–57. 2575769 10.1098/rstb.1989.0104

[11] Kalenga M, Tschanz SA, Burri PH (1995) Protein deficiency and the growing rat lung. II. Morphometric analysis and morphology. Pediatr Res 37: 789–795. 7651765 10.1203/00006450-199506000-00019

[12] Mortola JP (2001) Respiratory Physiology of Newborn Mammals: A Comparative Perspective The Johns Hopkins University Press,Baltimore, Maryland.

[13] Azzam MA, Mortola JP (2007) Organ growth in chicken embryos during hypoxia: implications on organ “sparing” and “catch-up growth”. Respir Physiol Neurobiol 159: 155–162. 17652035 10.1016/j.resp.2007.06.003

[14] Bartel DP (2004) MicroRNAs: genomics, biogenesis, mechanism, and function. cell 116: 281–297. 14744438 10.1016/s0092-8674(04)00045-5

[15] Filipowicz W, Bhattacharyya SN, Sonenberg N (2008) Mechanisms of post-transcriptional regulation by microRNAs: are the answers in sight? Nat Rev Genet 9: 102–114. 18197166 10.1038/nrg2290

[16] Jovanovic M, Hengartner MO (2006) miRNAs and apoptosis: RNAs to die for. Oncogene 25: 6176–6187. 17028597 10.1038/sj.onc.1209912

[17] Schickel R, Boyerinas B, Park SM, Peter ME (2008) MicroRNAs: key players in the immune system, differentiation, tumorigenesis and cell death. Oncogene 27: 5959–5974. 18836476 10.1038/onc.2008.274

[18] Barroso-Deljesus A, Lucena-Aguilar G, Sanchez L, Ligero G, Gutierrez-Aranda I, et al. (2011) The Nodal inhibitor Lefty is negatively modulated by the microRNA miR-302 in human embryonic stem cells. FASEB J. 10.1096/fj.10-172221 21266536

[19] Foshay KM, Gallicano GI (2007) Small RNAs, big potential: the role of MicroRNAs in stem cell function. Curr Stem Cell Res Ther 2: 264–271. 18220910 10.2174/157488807782793781

[20] Gunaratne PH (2009) Embryonic stem cell microRNAs: defining factors in induced pluripotent (iPS) and cancer (CSC) stem cells? Curr Stem Cell Res Ther 4: 168–177. 19492978 10.2174/157488809789057400

[21] Cimmino A, Calin GA, Fabbri M, Iorio MV, Ferracin M, et al (2005) miR-15 and miR-16 induce apoptosis by targeting BCL2. Proc Natl Acad Sci U S A 102: 13944–13949. 16166262 10.1073/pnas.0506654102 PMC1236577

[22] Yin KJ, Deng Z, Hamblin M, Xiang Y, Huang H, et al (2010) Peroxisome proliferator-activated receptor delta regulation of miR-15a in ischemia-induced cerebral vascular endothelial injury. J Neurosci 30: 6398–6408. 20445066 10.1523/JNEUROSCI.0780-10.2010 PMC2874744

[23] Pocock R (2011) Invited review: decoding the microRNA response to hypoxia. Pflugers Arch 461: 307–315. 21207057 10.1007/s00424-010-0910-5

[24] Lee RC, Ambros V (2001) An extensive class of small RNAs in Caenorhabditis elegans. science 294: 862–864. 11679672 10.1126/science.1065329

[25] Obernosterer G, Martinez J, Alenius M (2007) Locked nucleic acid-based in situ detection of microRNAs in mouse tissue sections. Nat Protoc 2: 1508–1514. 17571058 10.1038/nprot.2007.153

[26] Xu H, Wang X, Du Z, Li N (2006) Identification of microRNAs from different tissues of chicken embryo and adult chicken. FEBS Lett 580: 3610–3616. 16750530 10.1016/j.febslet.2006.05.044

[27] Piret JP, Mottet D, Raes M, Michiels C (2002) Is HIF-1alpha a pro- or an anti-apoptotic protein? Biochem Pharmacol 64: 889–892. 12213583 10.1016/s0006-2952(02)01155-3

[28] Greijer AE, van der Wall E (2004) The role of hypoxia inducible factor 1 (HIF-1) in hypoxia induced apoptosis. J Clin Pathol 57: 1009–1014. 15452150 10.1136/jcp.2003.015032 PMC1770458

[29] Cory S, Adams JM (2002) The Bcl2 family: regulators of the cellular life-or-death switch. Nat Rev Cancer 2: 647–656. 12209154 10.1038/nrc883

[30] Shimizu S, Eguchi Y, Kosaka H, Kamiike W, Matsuda H, et al (1995) Prevention of hypoxia-induced cell death by Bcl-2 and Bcl-xL. nature 374: 811–813. 7723826 10.1038/374811a0

[31] Calin GA, Cimmino A, Fabbri M, Ferracin M, Wojcik SE, et al (2008) MiR-15a and miR-16-1 cluster functions in human leukemia. Proc Natl Acad Sci U S A 105: 5166–5171. 18362358 10.1073/pnas.0800121105 PMC2278188

[32] Calin GA, Croce CM (2006) Genomics of chronic lymphocytic leukemia microRNAs as new players with clinical significance. Semin Oncol 33: 167–173. 16616063 10.1053/j.seminoncol.2006.01.010

[33] Grimson A, Farh KK, Johnston WK, Garrett-Engele P, Lim LP, et al (2007) MicroRNA targeting specificity in mammals: determinants beyond seed pairing. Mol Cell 27: 91–105. 17612493 10.1016/j.molcel.2007.06.017 PMC3800283

[34] Schuster P, Fontana W, Stadler PF, Hofacker IL (1994) From sequences to shapes and back: a case study in RNA secondary structures. Proc Biol Sci 255: 279–284. 7517565 10.1098/rspb.1994.0040

[35] Maragkakis M, Alexiou P, Papadopoulos GL, Reczko M, Dalamagas T, et al (2009) Accurate microRNA target prediction correlates with protein repression levels. BMC Bioinformatics 10: 295. 19765283 10.1186/1471-2105-10-295 PMC2752464

[36] Maragkakis M, Reczko M, Simossis VA, Alexiou P, Papadopoulos GL, et al (2009) DIANA-microT web server: elucidating microRNA functions through target prediction. Nucleic Acids Res 37: W273–276. 19406924 10.1093/nar/gkp292 PMC2703977

[37] Krek A, Grun D, Poy MN, Wolf R, Rosenberg L, et al (2005) Combinatorial microRNA target predictions. Nat Genet 37: 495–500. 15806104 10.1038/ng1536

[38] Lalthantluanga R, Wiesner H, Braunitzer G (1985) Studies on yak hemoglobin (Bos grunniens, Bovidae): structural basis for high intrinsic oxygen affinity? Biol Chem Hoppe Seyler 366: 63–68. 4005038 10.1515/bchm3.1985.366.1.63

[39] Zhang H, Burggren WW (2012) Hypoxic level and duration differentially affect embryonic organ system development of the chicken (Gallus gallus). Poult Sci 91: 3191–3201. 23155030 10.3382/ps.2012-02449

[40] Wang XY, He Y, Li JY, Bao HG, Wu C (2013) Association of a missense nucleotide polymorphism in the MT-ND2 gene with mitochondrial reactive oxygen species production in the Tibet chicken embryo incubated in normoxia or simulated hypoxia. Anim Genet 44: 472–475. 23347088 10.1111/age.12020

[41] Bao HG, Wang XY, Li JY, Wu CX (2011) Comparison of effects of hypoxia on glutathione and activities of related enzymes in livers of Tibet chicken and Silky chicken. Poult Sci 90: 648–652. 21325237 10.3382/ps.2010-00994

[42] Bao HG, Zhao CJ, Li JY, Zhang H, Wu C (2007) A comparison of mitochondrial respiratory function of Tibet chicken and Silky chicken embryonic brain. Poult Sci 86: 2210–2215. 17878451 10.1093/ps/86.10.2210

[43] ZHANG H, Wu C (2006) Influences of Oxygen on Embryonic Mortality and Hatchability of Chicken Eggs. Acta Veterinaria et Zootechnica Sinica 37(2): 112–116.

[44] Christensen VL, Bagley LG (1988) Improved hatchability of turkey eggs at high altitudes due to added oxygen and increased incubation temperature. Poult Sci 67: 956–960. 3413023 10.3382/ps.0670956

[45] Christensen VL, Bagley LG (1989) Efficacy of fertilization in artificially inseminated turkey hens. Poult Sci 68: 724–729. 2755899 10.3382/ps.0680724

[46] Christensen VL, Bagley RA (1984) Vital gas exchange and hatchability of turkey eggs at high altitude. Poult Sci 63: 1350–1356. 6473249 10.3382/ps.0631350

[47] Duncker HR (1971) The lung air sac system of birds. A contribution to the functional anatomy of the respiratory apparatus. Ergeb Anat Entwicklungsgesch 45: 7–171. 4258755

[48] King A (1989) Form and function in birds. London: Academic Press.

[49] Gallagher BC (1986) Basal laminar thinning in branching morphogenesis of the chick lung as demonstrated by lectin probes. J Embryol Exp Morphol 94: 173–188. 3760754

[50] Gallagher BC (1986) Branching morphogenesis in the avian lung: electron microscopic studies using cationic dyes. J Embryol Exp Morphol 94: 189–205. 3760755

[51] Maina JN (2003) A systematic study of the development of the airway (bronchial) system of the avian lung from days 3 to 26 of embryogenesis: a transmission electron microscopic study on the domestic fowl, Gallus gallus variant domesticus. Tissue Cell 35: 375–391. 14517104 10.1016/s0040-8166(03)00058-2

[52] Maina JN (2003) Developmental dynamics of the bronchial (airway) and air sac systems of the avian respiratory system from day 3 to day 26 of life: a scanning electron microscopic study of the domestic fowl, Gallus gallus variant domesticus. Anat Embryol (Berl) 207: 119–134. 12856178 10.1007/s00429-003-0333-6

[53] Liu C, Zhang LF, Song ML, Bao HG, Zhao CJ, et al (2009) Highly efficient dissociation of oxygen from hemoglobin in Tibetan chicken embryos compared with lowland chicken embryos incubated in hypoxia. Poult Sci 88: 2689–2694. 19903969 10.3382/ps.2009-00311

[54] Camps C, Buffa FM, Colella S, Moore J, Sotiriou C, et al (2008) hsa-miR-210 Is induced by hypoxia and is an independent prognostic factor in breast cancer. Clin Cancer Res 14: 1340–1348. 18316553 10.1158/1078-0432.CCR-07-1755

[55] Fasanaro P, D'Alessandra Y, Di Stefano V, Melchionna R, Romani S, et al (2008) MicroRNA-210 modulates endothelial cell response to hypoxia and inhibits the receptor tyrosine kinase ligand Ephrin-A3. J Biol Chem 283: 15878–15883. 18417479 10.1074/jbc.M800731200 PMC3259646

[56] Hebert C, Norris K, Scheper MA, Nikitakis N, Sauk JJ (2007) High mobility group A2 is a target for miRNA-98 in head and neck squamous cell carcinoma. Mol Cancer 6: 5. 17222355 10.1186/1476-4598-6-5 PMC1783857

[57] Kulshreshtha R, Ferracin M, Wojcik SE, Garzon R, Alder H, et al (2007) A microRNA signature of hypoxia. Mol Cell Biol 27: 1859–1867. 17194750 10.1128/MCB.01395-06 PMC1820461

[58] Oren M (2003) Decision making by p53: life, death and cancer. Cell Death Differ 10: 431–442. 12719720 10.1038/sj.cdd.4401183

[59] Vogelstein B, Kinzler KW (2004) Cancer genes and the pathways they control. Nat Med 10: 789–799. 15286780 10.1038/nm1087

[60] Yamakuchi M, Lotterman CD, Bao C, Hruban RH, Karim B, et al (2010) P53-induced microRNA-107 inhibits HIF-1 and tumor angiogenesis. Proc Natl Acad Sci U S A 107: 6334–6339. 20308559 10.1073/pnas.0911082107 PMC2851979

[61] Yue J, Guan J, Wang X, Zhang L, Yang Z, et al (2013) MicroRNA-206 is involved in hypoxia-induced pulmonary hypertension through targeting of the HIF-1alpha/Fhl-1 pathway. Lab Invest 93: 748–759. 23628900 10.1038/labinvest.2013.63

[62] Shan F, Li J, Huang QY (2014) HIF-1 Alpha-Induced Up-Regulation of miR-9 Contributes to Phenotypic Modulation in Pulmonary Artery Smooth Muscle Cells During Hypoxia. J Cell Physiol. 10.1002/jcp.24593 24615545

[63] Crosby ME, Kulshreshtha R, Ivan M, Glazer PM (2009) MicroRNA regulation of DNA repair gene expression in hypoxic stress. Cancer Res 69: 1221–1229. 19141645 10.1158/0008-5472.CAN-08-2516 PMC2997438

[64] Hayashita Y, Osada H, Tatematsu Y, Yamada H, Yanagisawa K, et al (2005) A polycistronic microRNA cluster, miR-17-92, is overexpressed in human lung cancers and enhances cell proliferation. Cancer Res 65: 9628–9632. 16266980 10.1158/0008-5472.CAN-05-2352

[65] Frankel LB, Christoffersen NR, Jacobsen A, Lindow M, Krogh A, et al (2008) Programmed cell death 4 (PDCD4) is an important functional target of the microRNA miR-21 in breast cancer cells. J Biol Chem 283: 1026–1033. 17991735 10.1074/jbc.M707224200

[66] Wang J, Haubrock M, Cao KM, Hua X, Zhang CY, et al (2011) Regulatory coordination of clustered microRNAs based on microRNA-transcription factor regulatory network. BMC Syst Biol 5: 199. 22176772 10.1186/1752-0509-5-199 PMC3262773

[67] Hua Z, Lv Q, Ye W, Wong CK, Cai G, et al (2006) MiRNA-directed regulation of VEGF and other angiogenic factors under hypoxia. PLoS One 1: e116. 17205120 10.1371/journal.pone.0000116 PMC1762435

[68] Yue J, Tigyi G (2010) Conservation of miR-15a/16-1 and miR-15b/16-2 clusters. Mamm Genome 21: 88–94. 20013340 10.1007/s00335-009-9240-3 PMC2820079

[69] Darnell DK, Kaur S, Stanislaw S, Konieczka JH, Yatskievych TA, et al (2006) MicroRNA expression during chick embryo development. Dev Dyn 235: 3156–3165. 17013880 10.1002/dvdy.20956

[70] Rasmussen KD, Simmini S, Abreu-Goodger C, Bartonicek N, Di Giacomo M, et al (2010) The miR-144/451 locus is required for erythroid homeostasis. J Exp Med 207: 1351–1358. 20513743 10.1084/jem.20100458 PMC2901075

[71] Sangokoya C, Telen MJ, Chi JT (2010) microRNA miR-144 modulates oxidative stress tolerance and associates with anemia severity in sickle cell disease. blood 116: 4338–4348. 20709907 10.1182/blood-2009-04-214817 PMC2993631

[72] Yang Y, Kai G, Pu XD, Qing K, Guo XR, et al (2012) Expression profile of microRNAs in fetal lung development of Sprague-Dawley rats. Int J Mol Med 29: 393–402. 22160159 10.3892/ijmm.2011.855

[73] Dong J, Jiang G, Asmann YW, Tomaszek S, Jen J, et al (2010) MicroRNA networks in mouse lung organogenesis. PLoS One 5: e10854. 20520778 10.1371/journal.pone.0010854 PMC2877109

[74] Mujahid S, Logvinenko T, Volpe MV, Nielsen HC (2013) miRNA regulated pathways in late stage murine lung development. BMC Dev Biol 13: 13. 23617334 10.1186/1471-213X-13-13 PMC3644234

[75] Bridges JP, Lin S, Ikegami M, Shannon JM (2012) Conditional hypoxia inducible factor-1alpha induction in embryonic pulmonary epithelium impairs maturation and augments lymphangiogenesis. Dev Biol 362: 24–41. 22094019 10.1016/j.ydbio.2011.10.033 PMC3262673

[76] Semenza GL (2014) Hypoxia-inducible factor 1 and cardiovascular disease. Annu Rev Physiol 76: 39–56. 23988176 10.1146/annurev-physiol-021113-170322 PMC4696033

[77] Tang R, Li L, Zhu D, Hou D, Cao T, et al (2012) Mouse miRNA-709 directly regulates miRNA-15a/16-1 biogenesis at the posttranscriptional level in the nucleus: evidence for a microRNA hierarchy system. Cell Res 22: 504–515. 21862971 10.1038/cr.2011.137 PMC3292299

[78] Zhang X, Chen X, Lin J, Lwin T, Wright G, et al. (2011) Myc represses miR-15a/miR-16-1 expression through recruitment of HDAC3 in mantle cell and other non-Hodgkin B-cell lymphomas. Oncogene. 10.1038/onc.2011.470 PMC3982396 22002311

[79] Wu G, Yu F, Xiao Z, Xu K, Xu J, et al (2011) Hepatitis B virus X protein downregulates expression of the miR-16 family in malignant hepatocytes in vitro. Br J Cancer 105: 146–153. 21629246 10.1038/bjc.2011.190 PMC3137408

[80] Cittelly DM, Das PM, Salvo VA, Fonseca JP, Burow ME, et al (2010) Oncogenic HER2{Delta}16 suppresses miR-15a/16 and deregulates BCL-2 to promote endocrine resistance of breast tumors. Carcinogenesis 31: 2049–2057. 20876285 10.1093/carcin/bgq192 PMC2994280

[81] Ofir M, Hacohen D, Ginsberg D (2011) MiR-15 and miR-16 are direct transcriptional targets of E2F1 that limit E2F-induced proliferation by targeting cyclin E. Mol Cancer Res 9: 440–447. 21454377 10.1158/1541-7786.MCR-10-0344

[82] Lin Q, Gao Z, Alarcon RM, Ye J, Yun Z (2009) A role of miR-27 in the regulation of adipogenesis. FEBS J 276: 2348–2358. 19348006 10.1111/j.1742-4658.2009.06967.x PMC5330386

[83] Iliopoulos D, Malizos KN, Oikonomou P, Tsezou A (2008) Integrative microRNA and proteomic approaches identify novel osteoarthritis genes and their collaborative metabolic and inflammatory networks. PLoS One 3: e3740. 19011694 10.1371/journal.pone.0003740 PMC2582945

[84] Ashburner M, Ball CA, Blake JA, Botstein D, Butler H, et al (2000) Gene ontology: tool for the unification of biology. The Gene Ontology Consortium. Nat Genet 25: 25–29. 10802651 10.1038/75556 PMC3037419

[85] Scheinfeldt LB, Tishkoff SA (2010) Living the high life: high-altitude adaptation. Genome Biol 11: 133. 20979669 10.1186/gb-2010-11-9-133 PMC2965377

