# Supplementary material for: Hypoxia-Induced miR-15a Promotes Mesenchymal Ablation and Adaptation to Hypoxia during Lung Development in Chicken
Source: PLoS One. 2014 Jun 2;9(6):e98868. doi: 10.1371/journal.pone.0098868 (PMC4041788; doi:10.1371/journal.pone.0098868)
Supplement: Table S1 — Reverse transcription primers and run method. (DOCX) [file pone.0098868.s001.docx]

Table S1. Reverse transcription primers and run method

| **gene symbol** | **GenBank(Gene ID)** | **reverse transcription primer** |
| --- | --- | --- |
| GAPDH | NM_204305 | oligo(dT) |
| *bcl-2* | 窗体顶端  NM_205339 窗体底端 | oligo(dT) |
| HIF-1 | NM_204297 | oligo(dT) |
| miR-144 | NR_031585 | 144R: 5′-CTCAACTGGTGTCGTGGAGTCGGCAATTCAGTTGAGGAGTACA-3′ |
| miR-15a | NR_031410 | 15aRT: 5′-CTCAACTGGTGTCGTGGAGTCGGCAATTCAGTTGAGACAAACC-3′ |
| miR-16 | NC_006088 | 16RT: 5′-CTCAACTGGTGTCGTGGAGTCGGCAATTCAGTTGAGCACCAATA -3′ |
| RN5S | NR_046276 | 5sRT: 5′-CTCAACTGGTGTCGTGGAGTCGGCAATTCAGTTGAGAAGCCTAC-3′ |

PCR run method：37℃ 2min; 25℃ 10min; 37℃ 50min; 70℃ 15min.
