# Supplementary material for: Hypoxia-Induced miR-15a Promotes Mesenchymal Ablation and Adaptation to Hypoxia during Lung Development in Chicken
Source: PLoS One. 2014 Jun 2;9(6):e98868. doi: 10.1371/journal.pone.0098868 (PMC4041788; doi:10.1371/journal.pone.0098868)
Supplement: Table S2 — Real-time PCR primers and run method. (DOCX) [file pone.0098868.s002.docx]

Table S2. Real-time PCR primers and run method

| **Gene Symbol** | **GenBank(Gene ID)** | **PCR Primer** |
| --- | --- | --- |
| GAPDH | NM_204305 | GAPDH forward primer: 5′-CGATCTGAACTACATGGTTTACATGTT-3′ |
|  |  | GAPDH reverse primer: 5′-CCCGTTCTCAGCCTTGACA-3′ |
| *bcl-2* | 窗体顶端  NM_205339 窗体底端 | bcl-2 forward primer: 5′-AGCGTCAACCGGGAGATGT-3′ |
|  |  | bcl-2 reverse primer: 5′-GCATCCCATCCTCCGTTGT-3′ |
| *HIF-1* | NM_204297 | HIF-1 forward primer: 5′-CAGGTACAA GAGCAACCAA CCA-3′ |
|  |  | HIF-1 reverse primer: 5′-TGGATAATGACATGGCTAATGAATTC-3′ |
|  |  | HIF-1 probe: 5′-FAM-AGTTCACCTGAGCCC-MGB-3′ |
| *miR-144* | NR_031585 | miR-144: 5′-CGCGGCTACAGTATAGATGATG-3′ |
|  |  | universal reverse primer: 5′-CTCAACTGGTGTCGTGGAGTC-3′ |
| *miR-15a* | NR_031410 | miR-15a: 5′-GCTGGTAGCAGCACATAATGG-3′ |
|  |  | universal reverse primer: 5′-CTCAACTGGTGTCGTGGAGTC-3′ |
| *miR-16* | NC_006088 | miR-16: 5′- GCGGAGTAGCAGCACGTAAA-3′ |
|  |  | universal reverse primer: 5′-CTCAACTGGTGTCGTGGAGTC-3′ |
| *RN5S* | NR_046276 | RN5S: 5′- GCTCTGGAATACCGGGTGCTGT-3′ |
|  |  | universal reverse primer: 5′-CTCAACTGGTGTCGTGGAGTC-3′ |

PCR run method：95℃ 10min; 95℃ 15s,60℃ 1min,(40 cycles).
