# Supplementary material for: Hypoxia-Induced miR-15a Promotes Mesenchymal Ablation and Adaptation to Hypoxia during Lung Development in Chicken
Source: PLoS One. 2014 Jun 2;9(6):e98868. doi: 10.1371/journal.pone.0098868 (PMC4041788; doi:10.1371/journal.pone.0098868)
Supplement: Table S3 — bcl-2 3′-UTR cloning primers. (DOCX) [file pone.0098868.s003.docx]

Table S3. *bcl-2* 3’-UTR cloning primers

| **Gene Symbol** | **GenBank(Gene ID)** | **PCR Primer** |
| --- | --- | --- |
| *bcl-2* | 窗体顶端  NM_205339窗体底端 | forward primer: 5′-CTCGAGAGTCACCCAGTTTATCGT-3′ |
|  |  | reverse primer: 5′-CTCGAGGATTCTTCCGCTTCGTCA-3′ |

PCR run method：94℃ 2min; 94℃ 30s,60℃ 30s,72℃ 1min(40 cycles); 72℃ 10min; 4℃ ∞.
